# Ventilatory Pattern Influences Tolerance to Normobaric Hypoxia in Healthy Adults

**DOI:** 10.3390/ijerph20064935

**Published:** 2023-03-10

**Authors:** Inés Albertus-Cámara, Cristina Rochel-Vera, Jose-Luis Lomas-Albaladejo, Vicente Ferrer-López, Ignacio Martínez-González-Moro

**Affiliations:** Research Group of Physical Exercise and Human Performance, Mare Nostrum Campus, University of Murcia, 30100 Murcia, Spain

**Keywords:** hypoxia, hypoxia tolerance test, controlled breathing, ventilatory, parachutists

## Abstract

Introduction: Tolerance to breathing in conditions with a decreased oxygen ratio is subject-specific. A normobaric hypoxia tolerance test (NHTT) is performed to assess the ability of each individual, as this may be influenced by genetic or personal factors such as age or gender. The aim of this study is to test the influence of deep breathing on hypoxia tolerance time. Material and methods: A total of 45 subjects (21 parachutists and 24 students) performed two NHTTs at 5050 m altitude (iAltitude). Arterial (SatO_2_) and muscle (SmO_2_) oxygen saturation were monitored with the Humon Hex^®^ device. The first NHTT was performed with free breathing, without any instructions; and the second NHTT was performed with wide, slow, diaphragmatic breathing. The NHTT was terminated at the end of 10 min or when a value of less than 83% was obtained. Results: The first NHTT was completed by 38.1% of parachutist and 33.3% of students while the second NHTT was completed by 85.7% and 75%, respectively. In the second NHTT, both parachutists and students had a significantly (*p* = 0.001) longer duration compared to the first NHTT. SmO_2_ and SatO_2_ values also increased significantly (*p* < 0.001) in both groups (*p* < 0.05). Conclusion: Performing controlled diaphragmatic breathing is successful in increasing hypoxia tolerance time and/or SatO_2_ values.

## 1. Introduction

Normobaric hypoxia presents air with reduced oxygen content without changing atmospheric pressure. It is a tool used in sports training [1] and in the recovery of various pathologies [2,3,4].

Another type of hypoxia is obtained during activities at high altitude above sea level, which is traditionally known as hypobaric hypoxia. In this type of hypoxia, the partial pressure of oxygen and atmospheric pressure decrease. Some sports disciplines are performed in these environmental conditions. Some examples are mountaineering, climbing or parachuting. Parachuting, in particular, may involve high altitude jumps called HALO (High Altitude Low Opening) and HAHO (High Altitude High Opening) in which the administration of exogenous oxygen is used [5].

The ability to breathe under these hypoxic conditions is referred to as hypoxia tolerance. Some factors are now known to influence hypoxia tolerance, such as genetics [6], age [7] and, in women, menstrual cycle timing, menopause or use of oral contraceptives [8]. Therefore, before designing a hypoxia training program for sports or rehabilitation purposes, a hypoxia tolerance test (HTT) should be performed.

Several biomarkers of susceptibility to hypoxia have been identified so far, such as hypoxia-inducible factor (HIF)-1 [9], heat shock protein 70 (HSP70) and, primarily, nitric oxide NO [10]. However, little is known about the influence of respiration type and how it can improve hypoxia tolerance. Breathing under reduced oxygen conditions can be a stressful stimulus for the organism. Traditionally, the effect of breathing on reducing states of anxiety or stress is well known [11], and hypoxia can sometimes be a stressful stimulus for the organism. Several oriental techniques, such as qigong or yoga [12], and more specifically pranayama, involve controlled breathing. The practice of pranayama has been shown to increase forced vital capacity, decrease systolic and diastolic blood pressure [13], improve arterial oxygen saturation [14] and reduce stress [15]. In this way, it may increase tolerance to hypoxia. One way to test how breathing influences hypoxia tolerance is by biofeedback of heart rate variability [16]. This type of biofeedback has been used as a control measure in other studies [17].

However, although the literature supports the benefits of controlled breathing in hypobaric hypoxia [18,19], the effect has never been tested using normobaric hypoxia in different populations. Normobaric hypoxia could constitute a more efficient and accessible resource for use in rehabilitation and sports training programs.

The aim of our study is to test the influence of controlled breathing on hypoxia tolerance in two different populations.

Our hypothesis is that, by performing controlled deep breathing, hypoxia tolerance time, arterial oxygen saturation and muscle oxygen saturation values will increase in both populations.

## 2. Material and Methods

### 2.1. Design

All aspects of this cross-over study design were carried out in the Biosanitary Research Laboratory (LAIB) of the University of Murcia. Consort guidelines for randomized clinical trials were followed [20]. This study received approval from the Research Ethics Committee of the University of Murcia (ID: 3657/2021), in accordance with the Declaration of Helsinki [21]. The participants signed an informed consent form and could leave the study at any time.

### 2.2. Participants

**Figure 1:**
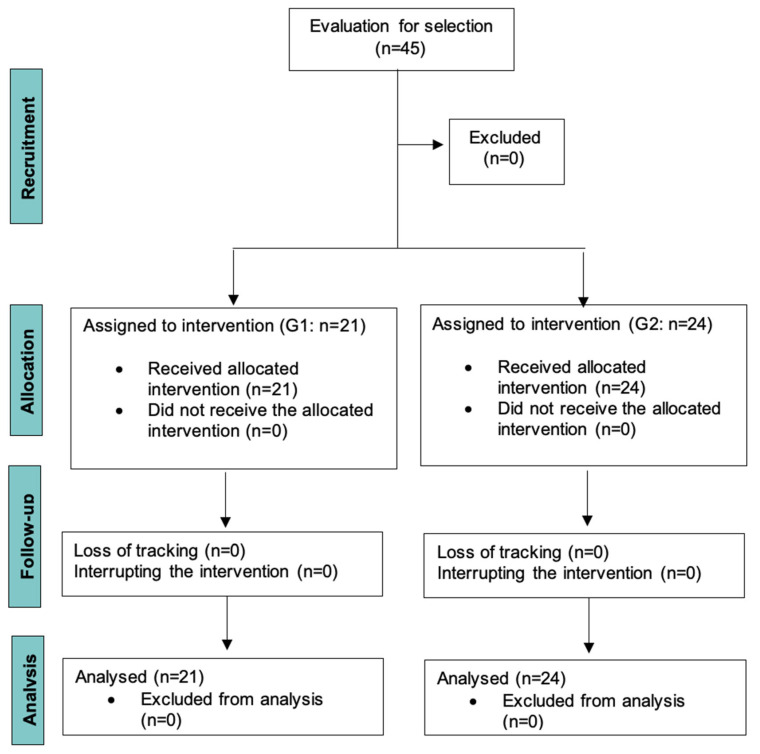
Flowchart of participants included in the study.

A total of 45 subjects made up the study: 21 professional parachutists with experience (G1) in high altitude jumps with the use of oxygen, and a second group composed of 24 healthy students (G2) with no experience in hypoxic activities (Figure 1). The parachutist group consisted of the Parachute Sapper Squadron (EZAPAC) and the Parachute Acrobatic Patrol of the Air and Space Army (PAPEA). The study consisted of two different groups to test the effect of controlled breathing on two different populations.

### 2.3. Criteria for Inclusion and Exclusion

The inclusion criterion for G1 was to be a professional skydiver with accredited experience in high altitude jumps that has performed hypoxic activities. For G2, healthy students with no experience in physical sports activities related to hypoxia (diving, parachuting, mountaineering, etc.) were required.

Those with cardiac and/or respiratory disorders that contraindicated hypoxia testing were excluded.

### 2.4. Outcome Measure

The measures analysed in the study were percentage arterial oxygen saturation (Nonin^®^ Ear Lobe Clip Sensor, Model 3018LP, Plymouth, MN, USA), percentage muscle oxygen saturation (Humon Hex^®^) and duration time in the hypoxia tolerance test (iAltitude^®^, Madrid, Spain). The pulse oximeter was placed in the participant’s left ear and the Humon-Hex device in the middle of the right quadriceps following the procedure described by Paredes et al. [22].

The simulated altitude was 5050 m, equivalent to an oxygen concentration of 11% (FiO_2_ = 0.11). The hypoxia simulator (iAltitude Trainer v2.7) had a tube connected to a specific mask through which oxygen-reduced air was circulated. This mask is specific to the hypoxia simulator, containing two valves that allow air to circulate in only one direction. Thus, air is always inhaled from the altitude simulator device and carbon dioxide is expelled to the outside. The hypoxia test was stopped when 10 min were reached or when the arterial oxygen saturation (SatO_2_) value was less than 83%. At that time, the hypoxia simulator emitted acoustic and visual signals indicating the removal of the respiratory mask under normoxic conditions. A cut-off value of 83% was determined by following the manufacturer’s recommendations.

The population was divided throughout the study into two groups: completes and incompletes. “Completes” are those subjects who managed to complete the maximum duration of the normobaric hypoxia tolerance test while “incompletes” are those subjects who did not complete the NHTT (SatO_2_ value dropped below 83%)

### 2.5. Preliminary Procedures

Before starting the NHTT, a preliminary examination was performed to check for the absence of cardiac or respiratory pathologies contraindicating the hypoxia test.

First, blood pressure was measured and the subject was auscultated (Littmann Classic III^®^, St. Paul, MN, USA). Next, an electrocardiogram (Cardioline Click^®^, Trento, Italy) and an echocardiogram (Clarius PA HD^®^, Vancouver, Canada) were performed. In this way, pathologies such as ventricular hypertrophy and regurgitant lesions of the aortic and mitral valves, among others, could be ruled out.

### 2.6. Hypoxia Tolerance Test

Participants were seated in an armchair during the two normobaric hypoxia tolerance tests. A lumbar backrest and a footrest were placed to maintain a correct and comfortable posture throughout the hypoxia test.

**Figure 2:**
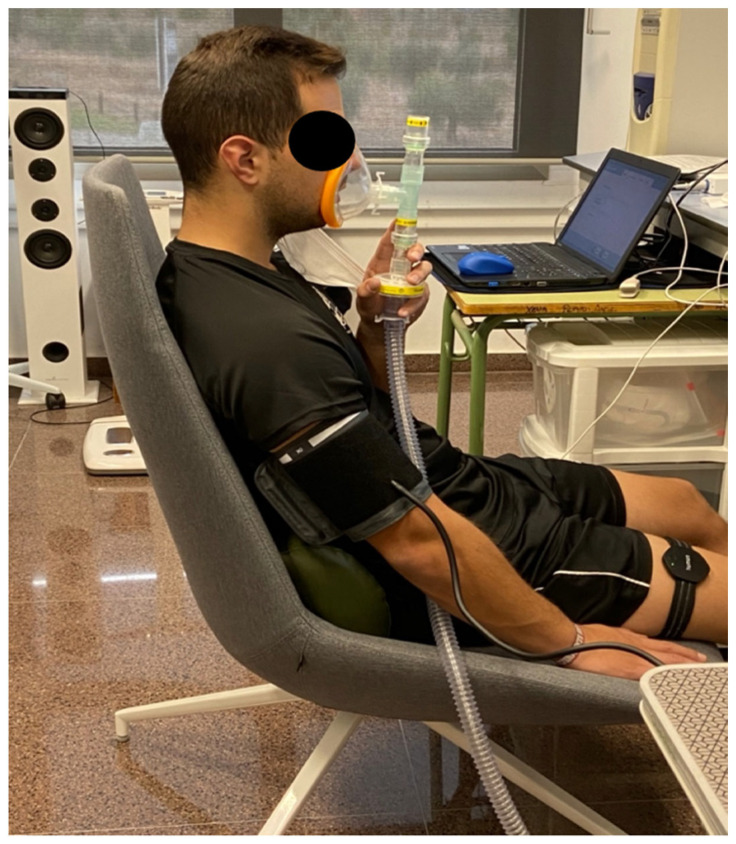
A subject performing the normobaric hypoxia tolerance test.

The participant held the specific mask over his or her face to breathe under hypoxic conditions (Figure 2). Directly in front of the subjects was a screen displaying the course of the NHTT. The device plotted a line according to SatO_2_ levels and another according to heart rate. The subject could see the evolution of each NHTT throughout its duration.

During both tests, arterial oxygen saturation and muscle oxygen saturation (SmO_2_) values were recorded.

Both NHTTs were performed under the same conditions and at the same altitude.

The difference between the first (NHTT1) and the second hypoxia test (NHTT2) lies in the way of breathing. In the first test, subjects maintained their usual, comfortable, unforced breathing rate. They were not given any instructions on how to breathe. After completing NHTT1, they spent 15 min breathing in normoxia before starting NHTT2.

To avoid the influence of instructions in the free breathing test (NHTT1), the order of testing was not randomized. Participants were unaware of the specifics of the test prior to the test.

**Figure 3:**
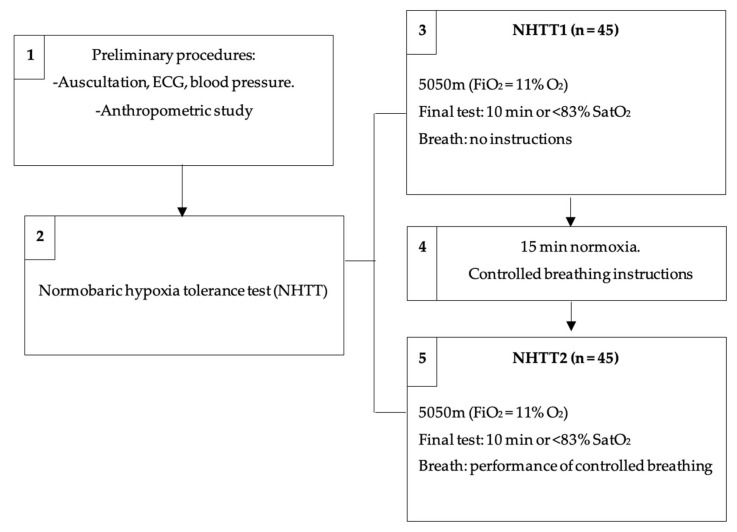
Outline of the phases of the investigation. ECG: electrocardiogram; NHTT: normobaric hypoxia tolerance test; FiO_2_: inspired oxygen fraction; NHTT1: first normobaric hypoxia tolerance test; NHTT2: second normobaric hypoxia tolerance test; SatO_2_: arterial oxygen saturation.

Before starting NHTT2, the type of breathing to be performed during this second hypoxia test was explained. It consisted of slow, wide, deep breathing with diaphragmatic breaths. A breathing rate of 8–10 breaths per minute was maintained. The subject practiced this breathing several times in normoxia under the supervision of the staff in charge. The volunteer then initiated NHTT2 while maintaining this breathing rate and amplitude. The investigator in charge of the test periodically reminded the subject of the breathing rate (Figure 3).

### 2.7. Data Analysis

After ruling out the presence of errors, the data were exported to the Statistical Package for Social Science (SPSSv.28^®^) to be analysed. Quantitative variables have been described with the mean and standard deviation (SD), and qualitative variables with absolute frequency and percentage. The normal distribution of the variables was verified using the Shapiro–Wilk test and the equality of variances using the Levene’s test. Comparison of means of independent intergroup variables was performed using Student’s t-tests, and comparison of means of related variables was made with paired t-tests. A X^2^ test (categorical variables) was used to analyse differences between groups. A minimum level of significance of *p* < 0.05 was established.

## 3. Results

### 3.1. Overall Assessment

A total of 45 subjects participated: expert parachutists (85.7% male) and healthy students (54.2% male). The parachutists had a mean of 2259 and a median of 1100 total jumps and 14.9 ± 10.3 average jumps performed with oxygen supply.

Table 1 shows the anthropometric characteristics separated by sex. Significant differences (*p* < 0.05) between groups (parachutists and students) are evident.

The physiological characteristics prior to the first exposure to hypoxia (baseline) are shown in Table 2, with no differences between groups, except for HR.

### 3.2. Duration of Hypoxia Tolerance Test

In the first test (NHTT1), of the 21 parachutists, eight (38.1%) completed the NHTT and of the 24 students, eight completed the NHTT (33.3%). There was no difference (χ^2^ = 0.111, *p* = 0.739) between having completed the test or not and belonging to one group or the other.

In the second test (NHTT2), the number of subjects who managed to complete the NHTT increased: 18 parachutists (85.7%) and 18 students (75%). There was also no difference between test completion and group (χ^2^ = 0.804, *p* = 0.370).

Table 3 shows this distribution by origin group.

### 3.3. Arterial and Muscle Oxygen Saturation in the NHTT1

Table 4 shows SatO_2_ and SmO_2_ separated by group and subgroup in the first tolerance test.

Among parachutists and students who did not complete NHTT1, differences (*p* < 0.05) were observed between the initial and final values of both values. In parachutists and students who did complete NHTT1, significant differences (*p* < 0.05) were observed between the initial and final values of SatO_2_, but not in muscle oxygen saturation in either subgroup.

On analysing the results of SatO_2_ and SmO_2_ in the second test in the same subgroups into which we divided the population (according to their completion of the first test), we observe, as shown in Table 5, that there are significant differences in the initial and final values of SatO_2_ in G1 (both in those who completed the test and those did not complete the test). In G2 this significant difference (*p* < 0.05) was only observed in those who did not complete the test, showing significantly higher values in SatO2 and SmO2. In the complete subgroup, no significant differences (*p* > 0.05) were observed between the initial and final values.

The values of arterial saturation, muscle saturation and time duration of NHTT1 and NHTT2 divided by groups are compared in Table 6. In both groups (G1 and G2) there are significantly (*p* < 0.05) higher values in NHTT2.

Table 7 shows the test duration, arterial oxygen saturation and muscle oxygen saturation in the first and second test by subgroup and group. In the subgroup of subjects who did not complete the NHTT test, there was a significant increase (*p* < 0.05) in time, muscle oxygen saturation and arterial oxygen saturation. On the other hand, those subjects who completed the NHTT showed significant differences (*p* < 0.05) in arterial saturation values. However, values of muscle oxygen saturation only improved in parachutists (G1)

**Figure 4:**
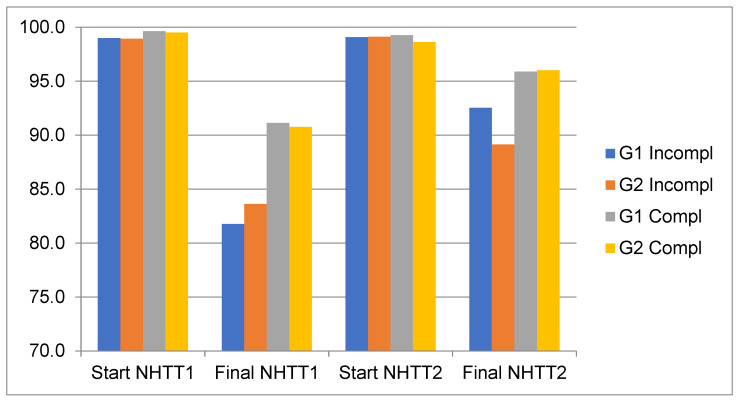
Evolution of SatO_2_ (%) in both test and subgroups. NHTT1: first normobaric hypoxia tolerance test; NHTT2: second normobaric hypoxia tolerance test; G1 Incompl: group of parachutists with incomplete test; G2 Incompl: group of students with incomplete test; G1 Compl: group of parachutists with complete test; G2 Compl: group of students with complete test.

Figure 4 shows the evolution of the SatO_2_ of both groups in the two tests. It can be seen that in both groups, the values of SatO_2_ at the beginning of the first test are similar, decreasing more at the end of the first test in those who did not complete the NHTT1. At the beginning of the second test, the values of the four subgroups are similar, but at the end of the second test the percentage of arterial saturation decreases less in all of them.

**Figure 5:**
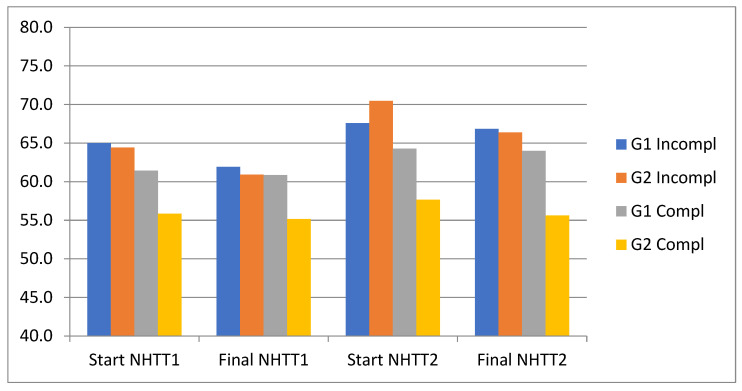
Evolution of SmO_2_ (%) in both test and subgroups. NHTT1: first normobaric hypoxia tolerance test; NHTT2: second normobaric hypoxia tolerance test; G1 incompl: group of parachutists with incomplete test; G2 Incompl: group of students with incomplete test; G1 Compl: group of parachutists with complete test; G2 Compl: group of students with complete test.

Figure 5 shows the evolution of SmO_2_ at the beginning and end of the first and second tests. It is evident that the values are similar at the beginning and decrease at the end of the first test for all four. A supercompensation effect is observed at the start of the second test, i.e., the values at the start of NHTT2 are higher than at the start of the first test. At the end of NHTT2 they decrease less than at the end of NHTT1.

## 4. Discussion

This study shows an increase in tolerance to normobaric hypoxia when controlled diaphragmatic breathing is performed in two different populations.

When volunteers perform controlled breathing (NHTT2) they perform better than when they perform free breathing (NHTT1). This may be because sympathetic activation caused by exposure to hypoxia [23] can be neutralized by controlled breathing, which is a popular and effective method of stress reduction [24]. Other authors have found that the inspiratory musculature decreases its fatigue by controlling the respiratory rate [25]. In addition, the type of breathing performed by the subject has different physiological effects. Slow, deep breathing has been found to result in increased oxygen uptake, increased tidal volume [26], increased arterial saturation and increased alveolar volume [27]. These findings demonstrate that slow, controlled breaths optimize arterial saturation values and, therefore, the subjects in our study showed increased tolerance to hypoxia in the second test.

Our study shows that this method of breathing is effective when subjects are exposed to an altitude of 5050 m (11% O_2_). However, other authors have shown that it is also effective at other altitudes. Nepal et al. [18] included two groups of subjects in their research. One group was exposed to an altitude of 2800 m and the second group an altitude of 3760 m. All subjects breathed deeply and slowly for four minutes in hypobaric hypoxia. The authors compared arterial saturation values and found that deep breathing improved the SatO_2_ value in both groups. Bilo et al. [19] exposed their volunteers to higher altitudes (4559 m and 5400 m) and also showed higher SatO_2_ values with this type of breathing. Our study, performed under normobaric hypoxia conditions, presents the same findings as these authors even with a longer exposure time.

In their study, Botella de Maglia et al. [28] analysed the influence of experience in hypoxic activities on arterial saturation values. They found that when mountaineers were exposed to hypoxic environmental conditions, they had higher SatO_2_ values than people without altitude experience. However, taking slow, deep breaths, such as those proposed in our study, improved adaptation to hypoxia in both groups in that study [28]. In our investigation, subjects who failed to complete NHTT1 improved hypoxia exposure time and arterial saturation values by taking controlled breaths in NHTT2. On the other hand, the subjects who did complete the first test managed to finish the second test with better SatO_2_ values. Thus, it is evident that this method is beneficial both for subjects with good initial adaptation to hypoxia and those with poor adaptation.

Adaptation to hypoxia is a characteristic of each subject, so in the study by Botek et al. [29] they divided the population into two groups: hypoxia-resistant subjects and hypoxia-sensitive subjects. This study used normobaric hypoxia and the same exposure time as our study (10 min). However, Botek et al. [29] only included men in their study, and the influence of sex on hypoxia tolerance is traditionally known [30]. Therefore, it would be interesting to conduct new studies that include more women. In this way, the findings obtained in our study could be consolidated.

The findings of this study have great applicability in the field of sports and in the rehabilitation of various pathologies. Skydiving is a risky activity, and in particular, high-altitude skydiving is an especially stressful activity for the athlete [31]. Performing these controlled breaths during a skydiver’s descent could increase hypoxia tolerance time and improve athletic performance. In addition, it could be a resource for emergency situations such as oxygen cylinder failure or disconnection, increasing the useful consciousness time [32] and even saving the skydiver’s life. Therefore, controlled breathing could be even more beneficial for people exposed to high-risk altitude sports.

Poor adaptation to hypoxia can lead to Acute Mountain Sickness Syndrome (AMS) [33]. As early as 1998, Roach et al. [34] demonstrated that subjects with hypoxemia at 4200 m at rest were at an increased risk of AMS. Following the design of our study, an NHTT could be performed to diagnose susceptibility and predict individual risk to hypoxic environments. In addition, the response to breathing control could be analysed.

Reduced respiratory rate results in increased cardiac–vagal baroreflex sensitivity (BRS) which is related to mental and physical health [35,36]. Impairment of the baroreflex mechanism occurs in conditions such as high blood pressure, diabetes, or cardiac infarction [37]. Our study may constitute a resource in rehabilitation programs. Patients in an acute state could perform this type of breathing and, when considered appropriate, include these breaths under hypoxic conditions. In this way, normobaric hypoxia could constitute a complementary technique in rehabilitation programs.

A limitation of our work is that we used normobaric hypoxia, which is a lower stimulus than hypobaric hypoxia. However, it is the most effective and realistic way to simulate altitude exposure under controlled and safe conditions.

## 5. Conclusions

Controlled diaphragmatic breathing at a high volume and slow rate improves hypoxia tolerance as measured by a normobaric hypoxia tolerance test at 5050 m altitude.

## Figures and Tables

**Table 1:** Anthropometric and body composition characteristics divided by sex and group.

Sex	Variable	Group	*n*	Mean	SD	t	*p*
Men	Age (years)	G1	18	39.2	8.6	7.423	0.000 *
		G2	13	22.7	3.4		
	Height (cm)	G1	18	178.17	7.19	2.084	0.023 *
		G2	13	173.38	4.81		
	Weight (kg)	G1	18	80.13	9.85	2.585	0.008 *
		G2	13	71.60	7.84		
	BMI (kg/m^2^)	G1	18	25.24	2.17	1.685	0.051
		G2	13	23.83	2.48		
	MGR (kg)	G1	18	20.69	3.02	1.462	0.077
		G2	13	18.65	4.78		
Women	Age (years)	G1	3	41.7	10.7	2.901	0.049 *
		G2	11	23.6	2.4		
	Height (cm)	G1	3	167.33	9.45	0.709	0.246
		G2	11	163.56	7.89		
	Weight (kg)	G1	3	66.33	15.30	0.977	0.213
		G2	11	57.54	5.75		
	BMI (kg/m^2^)	G1	3	23.47	2.87	1.224	0.122
		G2	11	21.57	2.27		
	MGR (kg)	G1	3	31.10	5.04	1.763	0.052
		G2	11	27.05	3.13		

MGR: relative fat mass; BMI: body mass index; G1: parachutists; G2: students. * *p* < 0.05.

**Table 2:** Basal physiological characteristics.

Variable	Group	Mean	SD	t	*p*
Heart rate (bpm)	G1	68.81	13.58	−2.159	0.018 *
	G2	77.92	14.57		
Systolic blood pressure (mmHg)	G1	124.48	14.06	−0.639	0.263
	G2	127.38	16.08		
Diastolic blood pressure (mmHg)	G1	82.00	9.83	0.687	0.248
	G2	80.04	9.29		
SatO_2_ (%)	G1	99.24	0.94	0.292	0.386
	G2	99.13	1.54		
SmO_2_ (%)	G1	63.68	6.02	0.599	0.276
	G2	61.56	14.21		

Bpm: beats per minute; mmHg: millimetres of mercury; SatO_2_: arterial oxygen saturation; SmO_2_: muscle oxygen saturation. * *p* < 0.05.

**Table 3:** Distribution of the population into subgroups according to completion of the test.

Test	Group				Total		
			Incomplete	Complete		χ^2^ Test	*p* Value
NHTT1	G1	Count	13	8	21	0.111	0.739
		% within supergroup	61.9%	38.1%	100.0%		
	G2	Count	16	8	24		
		% within supergroup	66.7%	33.3%	100.0%		
	Total	Count	29	16	45		
		% within supergroup	64.4%	35.6%	100.0%		
NHTT2	G1	Count	3	18	21	0.804	0.370
		% within supergroup	14.3%	85.7%	100.0%		
	G2	Count	6	18	24		
		% within supergroup	25.0%	75.0%	100.0%		
	Total	Count	9	36	45		
		% within supergroup	20.0%	80.0%	100.0%		

NHTT1: first normobaric hypoxia tolerance test; NHTT2_:_ second normobaric hypoxia tolerance test; G1: parachutists; G2: students. *p* < 0.05; χ^2^ test: chi-squared test.

**Table 4:** Differences in SatO_2_ and SmO_2_ in the first test separated by subgroup and group.

				Mean	SD	Paired *t*-test	*p* Value
G1	Incomplete (61.9%)	SatO_2_	Start	99.00	1.00	26.429	0.000 *
			Final	81.77	2.42		
		SmO_2_	Start	65.00	6.62	3.120	0.005 *
			Final	61.92	7.83		
	Complete (38.1%)	SatO_2_	Start	99.63	0.74	4.123	0.002 *
			Final	91.13	5.62		
		SmO_2_	Start	61.43	4.35	0.795	0.229
			Final	60.86	4.85		
G2	Incomplete (66.7%)	SatO_2_	Start	98.94	1.81	25.437	0.000 *
			Final	82.25	0.77		
		SmO_2_	Start	64.42	10.77	5.326	0.000 *
			Final	60.92	10.33		
	Complete (33.3%)	SatO_2_	Start	99.50	0.76	5.000	0.001 *
			Final	90.75	4.86		
		SmO_2_	Start	55.83	19.28	0.863	0.214
			Final	53.50	14.10		

G1: parachutists; G2: students; Incomplete: subjects who did not complete the NHTT1; Complete: subjects who did complete the NHTT1; SatO_2_: arterial oxygen saturation; SmO_2_: muscle oxygen saturation. * *p* < 0.05.

**Table 5:** Differences in SatO_2_ and SmO_2_ in the second test separated by group and subgroup.

Group	Subgroup	Variable	Mean		SD	Paired *t*-Test	*p* Value
G1	Incomplete (14.3%)	SatO_2_	Start	99.08	0.86	3.387	0.003 *
			Final	92.54	7.07		
		SmO_2_	Start	67.58	4.42	1.795	0.050
			Final	65.75	7.24		
	Complete (85.7%)	SatO_2_	Start	99.25	0.71	4.217	0.002 *
			Final	95.88	2.17		
		SmO_2_	Start	64.83	5.23	0.881	0.209
			Final	64.00	4.00		
G2	Incomplete (25%)	SatO_2_	Start	99.13	0.72	6.717	0.000 *
			Final	89.13	5.99		
		SmO_2_	Start	69.40	9.12	2.302	0.023 *
			Final	66.00	8.77		
	Complete (75%)	SatO_2_	Start	98.63	3.50	1.263	0.124
			Final	96.00	4.11		
		SmO_2_	Start	56.20	15.77	−1.773	0.075
			Final	58.40	13.35		

G1: parachutists; G2: students; Incomplete: subjects who did not complete the NHTT1; Complete: subjects who did complete the NHTT1; SatO_2_: arterial oxygen saturation; SmO_2_: muscle oxygen saturation. * *p* < 0.05.

**Table 6:** Differences between the first and second tests by groups.

Group	Variable	*n*	Mean	SD	Paired *t*-Test	*p* Value
G1	Time NHTT1	21	7.09	2.91	−3.655	0.001 *
	Time NHTT2	21	9.28	2.19		
	SatO_2_ NHTT1	21	85.33	6.02	−4.983	0.000 *
	SatO_2_ NHTT2	21	93.81	5.86		
	SmO_2_ NHTT1	18	61.78	6.86	−4.644	0.000 *
	SmO_2_ NHTT2	18	65.89	4.17		
G2	Time NHTT1	24	6.38	3.31	−3.537	0.001 *
	Time NHTT2	24	8.51	2.70		
	SatO_2_ NHTT1	24	86.00	5.69	−3.560	0.001 *
	SatO_2_ NHTT2	24	91.42	6.28		
	SmO_2_ NHTT1	16	57.94	12.51	−5.375	0.000 *
	SmO_2_ NHTT2	16	63.00	10.69		

G1: parachutists; G2: students; NHTT1: first normobaric hypoxia tolerance test; NHTT2: second normobaric hypoxia tolerance test; SatO_2_: arterial oxygen saturation; SmO_2_: muscle oxygen saturation. * *p* < 0.05.

**Table 7:** Differences between the first and second test divided into subgroups and groups.

Subgroup	Group	Variable	*n*	Mean	SD	Paired *t*-Test	*p* Value
Incomplete	G1	Time NHTT1	13	5.30	2.23	−4.689	0.000 *
		Time NHTT2	13	8.84	2.73		
		SatO_2_ NHTT1	13	81.77	2.42	−4.731	0.000 *
		SatO_2_ NHTT2	13	92.54	7.07		
		SmO_2_ NHTT1	12	61.92	7.83	−4.088	0.001 *
		SmO_2_ NHTT2	12	66.83	4.22		
	G2	Time NHTT1	16	4.58	2.51	−4.100	0.000 *
		Time NHTT2	16	7.76	3.07		
		SatO_2_ NHTT1	16	83.63	4.53	−2.673	0.009 *
		SatO_2_ NHTT2	16	89.13	5.99		
		SmO_2_ NHTT1	11	60.64	10.79	−5.885	0.000 *
		SmO_2_ NHTT2	11	66.36	8.92		
Complete	G1	SatO_2_ NHTT1	8	91.13	5.62	−2.357	0.025 *
		SatO_2_ NHTT2	8	95.88	2.17		
		SmO_2_ NHTT1	6	61.50	4.97	−2.712	0.021 *
		SmO_2_ NHTT2	6	64.00	3.69		
	G2	SatO_2_ NHTT1	8	90.75	4.86	−2.430	0.023 *
		SatO_2_ NHTT2	8	96.00	4.11		
		SmO_2_ NHTT1	5	52.00	15.22	−1.668	0.085
		SmO_2_ NHTT2	5	55.60	11.39		

Incomplete: subjects who did not complete the NHTT1; Complete: subjects who did complete the NHTT1; G1: parachutists; G2: students; NHTT1: first normobaric hypoxia tolerance test; NHTT2: second normobaric hypoxia tolerance test; SatO_2_: arterial oxygen saturation; SmO_2_: muscle oxygen saturation. * *p* < 0.05.

## Data Availability

Not applicable.
